# Sensitivity of RSV detection by PCR in respiratory samples is not reduced by a 24 h delay from sampling to testing with storage at room temperature

**DOI:** 10.1186/s10020-025-01403-2

**Published:** 2025-11-22

**Authors:** Katie Lihou, Serena McGuinness, Begonia Morales-Aza, Elizabeth Begier, Kaltun Duale, Rosa Aldridge, Nellie Farhoudi, Jonathan Vowles, Dylan Thomas, Jo Southern, Jennifer Oliver, Maria Lahuerta, Kathy Schneider, Bradford D. Gessner, Adam Finn, Leon Danon, Catherine Hyams, The AvonCAP Research Group, Aaran Sinclair, Ainhoa Rodriguez-Pereira, Amelia Langdon, Amy Taylor, Anabella Turner, Anna Jones, Anna Koi, Anya Mattocks, Azizah Azis, Ben Hitchings, Bethany Osborne, Brianna Dooley, Callum Hawkins, Caye Lisondra, Charles Butler, Charli Grimes, Chloe Farren, Christian Povey, Claire Mitchell, David Adegbite, Elinor Balch, Ella Ackroyd-Weldon, Emma Bridgeman, Emma Scott, Felix Wright, Ffion Davies, Fiona Perkins, Francesca Bayley, Gabriella Ruffino, Gabriella Valentine, Ged Ruffell, Georgina Mortimer, Grace Tilzey, Harriet Ibbotson, Harriet Kempson, Hanah Batholomew, Hephzibah Robb, Hugo Swift, Jacob Symanowski, Ilana Kelland, Imogen Ely, Jade King, Jake Whittle, James Conner, Jane Kinney, Jason Harkness, Johanna Kellett Wright, Josephine Bonnici, Josh Anderson, Juan Garcia-Tello, Julia Brzezinska, Julie Cloake, Kajal Naukariya, Katarina Milutinovic, Kate Helliker, Katie Maughan, Katy Tong, Kazminder Fox, Kellie Pettinger, Konstantina Minou, Kyla Chandler, Lana Ward, Leah Fleming, Leigh Morrison, Liberty Smith, Lily Smart, Lisa Grimmer, Louise Setter, Louise Wright, Lucy Grimwood, Maddalena Bellavia, Madeleine Clout, Maia Lyall, Malak Eghleilib, Marianne Vasquez, Maria Garcia Gonzalez, Mariella Ardeshir, Marta Mergulhao, Martina Chmelarova, Matthew Randell, Michael Booth, Milo Jeenes-Flanagan, Miriama Resutikova, Monika Chaulagain, Natalie Chang, Nefeli Tavira, Niall Grace, Nicola Manning, Nicolas Daryell-Armes, Nyomie Brown, Oliver Griffiths, Olivia Pearce, Pip Croxford, Peter Sequenza, Petronela Anchidin, Phoebe Kibble, Rajeka Lazarus, Rebecca Clemence, Rhian Walters, Riley Cooper, Robin Marlow, Robyn Heath, Rosie Newman-Hopkins, Rupert Antico, Sam Kemery, Sandi Nammuni Arachchge, Sarah Stollery, Seevakumar Suppiah, Sean Robinson, Siddiqa Uddin, Taslima Mona, Tawassal Riaz, Teagan Barrett, Tom Long, Tom Nyamunda, Tudor Dimofte, William Healy, Yassin Ben Khoud, Vicki Mackay, Zahra Hashmi, Zandile Maseko, Zoe Taylor, Zsuzsa Szasz-Benczur, Zsolt Friedrich

**Affiliations:** 1 Bristol Vaccine Centre, Schools of Population Health Sciences & Cellular and Molecular Medicine, University of Bristol, St Michael’s Hill, Bristol, BS2 8AE UK; 2 Engineering Mathematics, University of Bristol, Bristol, UK; 3 Global Respiratory Vaccines, Medical & Scientific Affairs, Pfizer Inc, Collegeville, PA USA; 4 Medical Evidence Generation, Pfizer Inc, Collegeville, PA USA

**Keywords:** RSV incidence, PCR, ALRTD diagnosis, Viral testing, Sample stability

## Abstract

Respiratory Syncytial Virus (RSV) is a common cause of severe respiratory tract disease in infants, the elderly and immunocompromised patients. However, there is uncertainty as to how sample handling practices affect performance of tests to detect RSV. The aim of this study was to determine whether RSV RNA remains reliably detectable in nasopharyngeal/oropharyngeal (NP/OP) samples, saliva, and sputum samples over time.

Respiratory samples were collected as part of a prospective observational study of acute lower respiratory tract disease (aLRTD) hospitalisations in adults in Bristol (UK). Samples that tested positive by PCR on receipt (0 h), were re-tested at 24 h having been stored at room temperature. We found that all but one of the samples PCR-positive for RSV at 0 h remained positive at 24 h, across all sample types and RSV strains. Ct values for NP/OP and saliva samples were significantly lower at 24 h than at 0 h, suggesting potential low-level viral replication in the samples. These results suggest that RSV tests can provide consistent results after a delay of up to 24 h following sample collection.

## Introduction

Tests undertaken for respiratory infection need to be accurate to guide clinical management decisions and facilitate surveillance and epidemiological studies. Respiratory Syncytial Virus (RSV) is a common cause of severe respiratory tract disease in infants, the elderly and immunocompromised patients (Langley & Anderson 2011; Savic et al. 2023). However, there is uncertainty as to how sample handling practices affect outcomes of PCR tests used to detect the virus, and thus diagnostic accuracy.

RSV is a single-stranded, negative-sense, non-segmented RNA virus, which is enveloped, pleomorphic and highly contagious. However, the virus is considered vulnerable to environmental changes, particularly temperature and humidity. The recovery of RSV from growth media and nasal secretions is reported to decrease over time on environmental surfaces, with loss of detection after 8 h from inoculation onto these surfaces (Breese et al., 1980). Another study reported approximately 10% survival of RSV on drying in 1 μl drops of tissue culture homogenate on polythene and approximately 1% survival after 24 h, differing somewhat with different media and humidity conditions, but showed no significant loss in liquid suspension over 3 h (Kingston 1968). Notably, these early studies required that the virus be cultivatable. Investigation of the structural stability of RSV, using spectroscopic and light scattering techniques, found RSV to be stable until temperatures of 40–50 °C at neutral pH, but had lower thermal stability in more acidic conditions (Ausar et al., 2005). None of these studies were investigating detection by polymerase chain reaction (PCR).

When collected from patients, respiratory specimens may be left at the bedside or in transit at ambient temperature for prolonged and uncontrolled periods of time before reaching laboratory testing facilities. Consequent degradation of RSV could result in false negative test results, but the degree to which this occurs has not previously been evaluated. The aim of this study therefore was to determine whether RSV remains detectable by PCR in nasopharyngeal/oropharyngeal (NP/OP), saliva, and sputum samples from patients hospitalised with acute respiratory conditions when held at room temperature for 24 h.

## Methods

### Data collection

Respiratory samples were obtained as part of an existing observational study, the Avon Community Acquired Pneumonia (AvonCAP) study conducted in Bristol (UK), which collected prospective data on acute lower respiratory tract disease (aLRTD) hospitalisations, including RSV infection. For full study details and methodology, see Hyams et al. (2022); this study recruited adults (aged ≥ 18y) hospitalised with signs/symptoms of acute lower respiratory tract disease in both hospitals in Bristol. Samples collected during RSV seasons in 2021–2022, 2022–2023, and 2023–2024 were included, with participants providing one or more an upper respiratory sample (nasopharyngeal or combined nasopharyngeal/oropharyngeal swab (NP/OP)), saliva, or a sputum sample. If individuals were unable to produce saliva, a saline mouth wash specimen was collected.

### Sample processing and testing

Samples were kept and transported at room temperature to the laboratory and were processed within 4 h of collection. Saliva and sputum samples were stored neat. NP/OP swab samples were stored in skim-milk, tryptone, glucose, and glycerin (STGG) broth medium (O'Brien, et al., 2001); Turner et al. 2011), which was the viral transport media included in the swab collection kit. Immediately after arrival in the laboratory, each specimen was split into two equal aliquots with an initial aliquot for PCR analysis was either taken immediately after laboratory processing (within 4 h of collection but from here on referred to as the 0-h sample), or after a freeze–thaw cycle, and stored at −70 °C. A second aliquot was used for the 24-h sample; with this aliquot being stored at room temperature (standard UK laboratory temperature is between 18 °C and 24 °C) for 24 h before freezing. Each paired 0-h and 24-h sample received the same number of freeze–thaw cycles (87% underwent a single freeze thaw cycle before analysis while the remaining 13% were not freeze thawed). This paired split-aliquot design to isolate the effect of ambient hold time. If samples were RSV test-positive on PCR at '0 h', the corresponding 24-h aliquots were also tested. RSV test-positive samples at ‘0 h’ and the corresponding 24-h samples were further analysed using an RSV A/B specific PCR test to determine the subgroup classification; RNA purification and detection was conducted separately for time 0 h and 24 h samples.

Samples were tested for RSV using VIASURE Real-Time PCR Detection Kits (CerTest Biotec S.L.) following the manufacturer's instructions. The VIASURE Respiratory Viral Panel I Kit was used for the identification of RSV, and the VIASURE RSV A + B Real Time PCR Detection Kit was used for subtyping. Viral targets were detected through one-step reverse transcription quantitative real-time PCR (RT-qPCR) using the QuantStudio 7 Flex Real-Time PCR System (Applied Biosystems). PCR results were analysed and exported using Design & Analysis v2.6.0 (Thermo Fisher Scientific), with the same detection threshold set for RSV and its A/B subtypes.

### Statistical methods

All analyses were conducted in R v4.3.3 (R Core Team 2021). Data were descriptively summarised, and 95% confidence intervals were calculated using the Clopper-Pearson method. A linear mixed effects model was used to test for differences in Cycle threshold (Ct) values between sample testing points (0 h; 24 h) for each sample type. Season was included as a random effect to control for differences between the winter seasons when samples were collected (2021–2022; 2022–2023; 2023–2024), and participant ID was included as a random effect to account for the paired sampling design (Ct ~ Time + (1| Season) + (1| Participant ID)). The model was run using the ‘lmerTest’ package which calculated *P* values using Satterthwaite's method for denominator degrees of freedom and t-statistics (Kuznetsova et al. 2017).

## Results

Overall, 113 paired samples tested positive for RSV by PCR at 0 h, and were re-tested at 24 h. Of these paired samples, 62 (54.9%) were NP/OP samples, 28 (24.8%) were sputum samples and 23 (20.4%) were saliva samples. The samples were from 90 different participants, of whom 15 provided two sample types, and four provided all three sample types. Of the 113 paired samples, 19 (16.8%) were from the 2021–2022 winter season, 61 (54.0%) were from the 2022–2023 winter season, and 33 (29.2%) were from the 2023–2024 winter season. At 0 h, 52 (46.0%) samples were RSV A positive only, 56 (49.6%) were RSV B positive only, and 5 (4.4%) were positive for both.

**Fig. 1:**
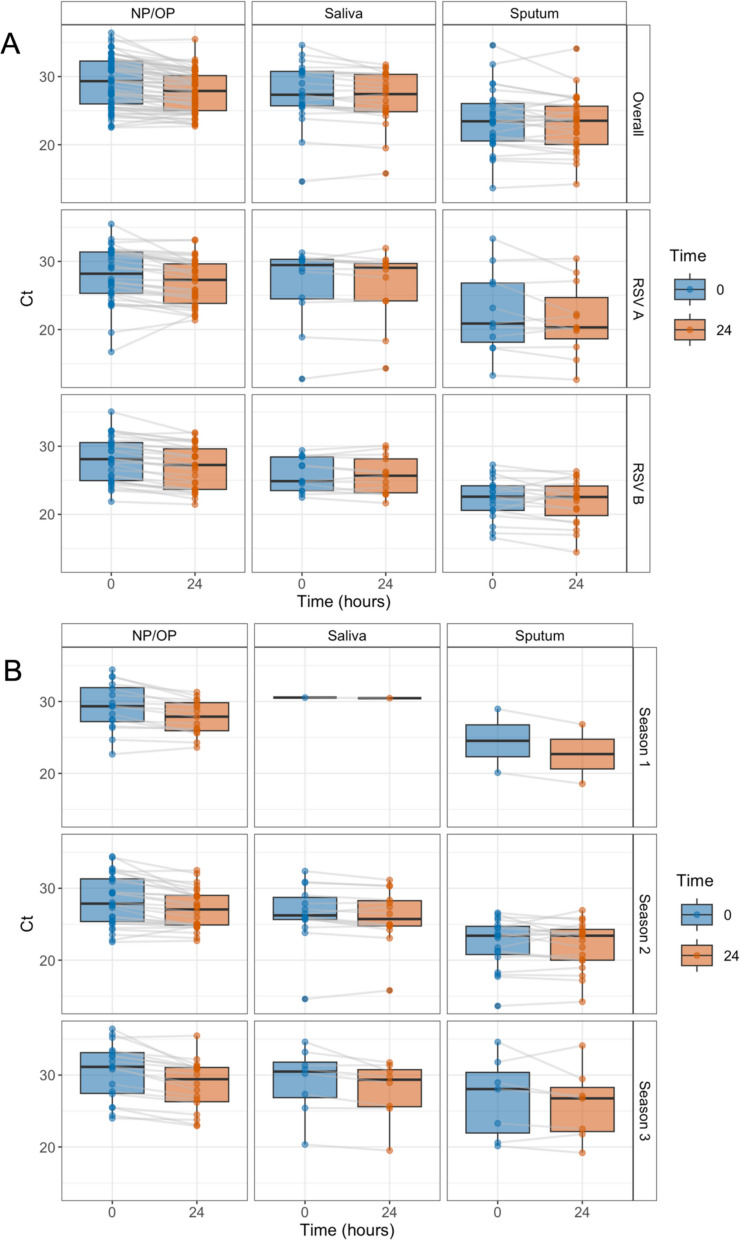
Sample Ct values with sampling to testing delay among samples testing RSV-positive at ‘0-h'. Boxplots of Ct values from PCR testing for RSV at sample collection (0 h; blue) and after the sample had been left at room temperature and re-tested (24 h; orange), split by sample type (NP/OP; saliva; sputum), and by (**A**) RSV type (overall non-strain specific RSV test result; RSV A test result; RSV B test result), and (**B**) collection year (Season 1 = 2021–2022; Season 2 = 2022–2023; Season 3 = 2023–2024). Paired data are connected by the grey lines. The middle line in the box represents median, while the box represents the interquartile range

Only one sample that was PCR positive at 0 h was not PCR positive at 24 h on the viral panel test, although it remained positive for RSV A on the specific subgroup A/B PCR test at 24 h. All positive samples had the same RSV strain(s) detected at 0 and 24 h (Table 1). This suggests that that any degradation to the 0 h samples caused by freezing did not result in further degradation to undetectable levels in the 24 h samples. The strain non-specific RSV PCR assay showed lower Ct values for NP/OP samples at 24 h than at 0 h (Est = −1.36; SE = 0.18; df = 59.96; t = −7.73; *p* < 0.0001) and saliva samples (Est = −0.72; SE = 0.22; df = 22.00; t = −3.30; *p* = 0.0032), but there was no difference for sputum samples (Est = −0.53; SE = 0.33; df = 27.00; t = −1.61; *p* = 0.1191) (Fig. 1; Supplementary Table 1).

**Table 1:** Sample concordance with sampling to testing delay among samples testing RSV-positive at ‘0 h’. The number of RSV test positives when tested at 0 and 24 h from sample collection, and percentage concordance in the number of positives split by upper respiratory swab sample type (Naso- or oropharyngeal (NP/OP); Saliva; Sputum) and RSV type (Overall (undefined)/A/B). Binomial 95% confidence intervals are Clopper-Pearson

Sample type	RSV type	No. positive: 0 h	No. positive: 24 h	Percentage concordance (95% CI)
NP/OP	Overall	62	61	98.4 (91.3–100.0)
	RSV A	34	34	100.0 (89.7–100.0)
	RSV B	29	29	100.0 (88.1–100.0)
Saliva	Overall	23	23	100.0 (85.2–100.0)
	RSV A	12	12	100.0 (73.5–100.0)
	RSV B	13	13	100.0 (75.3–100.0)
Sputum	Overall	28	28	100.0 (87.7–100.0)
	RSV A	11	11	100.0 (71.5–100.0)
	RSV B	19	19	100.0 (82.4–100.0)

## Discussion

This study evaluated the detection of RSV RNA by PCR in NP/OP, saliva, and sputum samples held at room temperature for 24 h. We found that all but one of the 113 samples which were RSV PCR-positive at 0 h remained positive at 24 h, across all sample types and RSV strains. The one sample that tested negative at 24 h did test positive on the strain-specific PCR. These findings suggest that delays in sample processing of up to 24 h at ambient temperatures do not result in significant numbers of false negatives for the RSV PCR testing methods used in this study, as was suggested by previous studies of the stability of RSV under various environmental conditions (Kingston 1968; Breese et al., 1980). Taken together, these findings indicate that delayed processing at ambient temperature does not materially reduce *PCR* detection of RSV, but they do not establish the stability of infectious virus, which is known to be environmentally labile under many conditions (Collins 2007).

Surprisingly, Ct values for many samples were lower at 24 vs 0 h across all sample types; this effect was statistically significant for NP/OP and saliva samples. Lower Ct values represent fewer PCR cycles required to detect RSV, indicating higher viral RNA concentrations in the tested samples. This in turn indicates that RSV RNA concentration did not decay exponentially, as might be expected in samples in the absence of cells (Beauchemin et al. 2019), suggesting that other factors prevented or balanced out RSV degradation in the samples. This could be due to breakdown in the protein capsid, increasing the concentration of accessible RNA, but it is also possible that viral replication continued to occur in the sample when held at room temperature and this mechanism could act to increase test sensitivity over time. Kitai et al. (2022) found that RSV from clinical samples (nasal swabs, throat swabs, nasal aspirates) stored at 20 °C retained infectivity after 24 h and that more characterised strains remained infectious at 20 °C than at 4 °C, although the mechanism behind this is unknown. Yamamoto et al. (2024) found a longer duration of RSV stability when stored at 4 °C than reported by Kitai et al. (2022) and suggest that this difference was due to the different sample media used (Viral Transport Medium and minimum essential medium, respectively). Castriciano et al. (2005) found that RSV stored at room temperature (20 °C) was viable after 96-h when stored in UTM-RT (universal transport medium), but only up to 48 h when stored in M4-RT (MicroTest) medium. Overall, these studies suggest that RSV may retain the ability to replicate in laboratory conditions, depending on sample media and temperature. In our study, the fall in Ct values over 24 h was greatest in NP/OP samples, with a modelled difference of −1.36 (SE 0.18), equivalent to a × 2.57 increase in target RNA concentration. This increase could reflect viral replication occurring in epithelial cells present in the samples. Differing relative concentrations of epithelial cells and mucus containing mucins and microbicidal enzymes, which can inhibit infection of epithelial cells (Chatterjee et al. 2020), could explain differing levels of replication in different samples and sample types. The room in which the samples were held for 24 h was air-conditioned but not closely monitored, and temperature differences may also have affected RNA concentration changes in different samples. Additionally, saliva and sputum samples were not stored in a culture medium, whereas the NP/OP samples included STGG broth which may have reduced RNA degradation through the stabilising effects of medium components, such as sugars (Law and Hull 1968). The increases in RSV RNA concentration over 24 h observed in this study were small compared to increases observed in vitro in cell culture – a meta-analysis of in vitro RSV studies, modelled an average growth rate of 0.18UI/h, equivalent to a ~ × 75 increase in RNA concentration over 24 h (Gonzàlez-Parra et al. 2018). Further studies in controlled conditions are needed to quantify in more detail the impact of sample type, storage media and physical conditions on changes in RSV RNA concentrations in respiratory samples over time.

Our study should be interpreted in the context of the following limitations. RSV detection concordance between time-points will vary with the relative sensitivity and specificity of the testing methods used, so should be interpreted in this context. The PCR assays used in this study had lower sensitivity and higher specificity for RSV detection compared to a clinical diagnostic laboratory standard-of-care testing-platform (Lihou et al. 2025) thus may only have detected RSV in samples with relatively high viral concentrations but would also have been therefore less likely to return false positives. If more sensitive testing methods were used, samples initially testing positive but with low viral concentrations might be more likely to degrade to undetectable levels within 24 h. As sensitivity is a trade off with specificity, more sensitive tests would also report more false positives at both 0 and 24 h, but not necessarily in the same samples, therefore falsely resulting in lower concordance between the two timepoints. As well as the testing platform used, different sample types also have different sensitivities documented for the detection of RSV in adults (Falsey et al. 2012), and this may also affect the agreement between initial and subsequent detection. We did not re-test samples that were negative at time 0 h, so only present findings on the consistency of initially positive results. Of note, this study assessed the impact of testing delay, not sampling delay. Delaying specimen collection could reduce test sensitivity in adults, likely due to lower viral loads in respiratory secretions among adults than children, which can then reduce more quickly to non-detectable levels during the disease course (Walsh et al. 2013). While samples were kept at room temperature in an air-conditioned laboratory, with a standard temperature between 18 °C and 24 °C, we did not closely monitor the temperature during this experiment, and thus this may affect our results. We did not test a time point beyond 24 h, and future work should randomize NP/OP, saliva, and sputum samples into commonly used media (eg, UTM/UTM-RT, M4-RT, dry swab with stabilization buffer) and test PCR positivity/Ct shift over 0, 2, 3, 5, and 7 days at 20–25 °C with heat-excursion controls, to directly inform mail-in protocols.

In summary, this study found no reduction in RSV PCR detection levels for NP/OP, saliva and sputum samples left at ambient temperature for 24 h vs at 0 h, suggesting that results from samples tested after a delay following sample collection still provide consistent information about RSV status, and that RSV burden estimates in adults hospitalised with aLRTD cannot a priori be assumed to be underestimated due to such testing delays. However, due to the different factors that impact test error rates, including testing platform and sample type, testing delays may have study-specific impacts on RSV burden estimates.

## Data Availability

The data used in this study are sensitive and cannot be made publicly available without breaching patient confidentiality rules. Therefore, individual participant data and a data dictionary are not available to other researchers.
